# Ethyl 2,4-dimethyl­pyrido[1,2-*a*]benz­imidazole-3-carboxyl­ate

**DOI:** 10.1107/S1600536812040299

**Published:** 2012-09-29

**Authors:** Ai Guo Zhu, Zhuo Ming Ma, Lin Jie Li, Tian Tian Wei, Yan Qing Ge

**Affiliations:** a School of Chemistry and Chemical Engineering, Taishan Medical University, Tai an 271016, People’s Republic of China

## Abstract

The title compound, C_16_H_16_N_2_O_2_, was synthesized using a novel tandem annulation reaction between 1-(1*H*-benzo[*d*]imidazol-2-yl)ethanone and ethyl (*E*)-4-bromo­but-2-enoate under mild conditions. The dihedral angles formed by the mean plane of the five-membered imidazole ring with the dihydro­pyridin and benzene rings are 1.54 (9) and 1.85 (9)°, respectively.

## Related literature

For the synthesis and characterization of pyrido[1,2-*a*]benz­imidazole derivatives, see: Ge *et al.* (2009 ▶, 2011 ▶). For pharmaceutical applications of nitro­gen-containing heterocyclic compounds, see: Badawey & Kappe (1999 ▶).
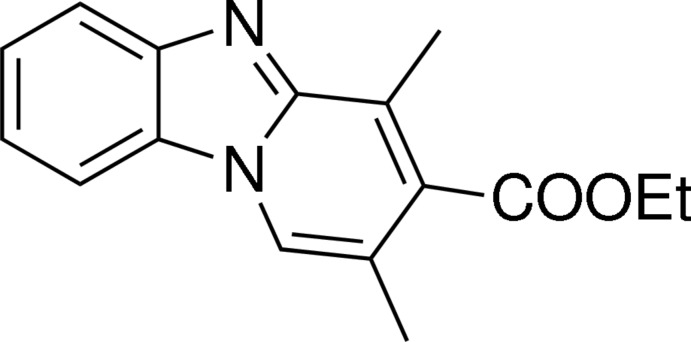


## Experimental

### 

#### Crystal data

C_16_H_16_N_2_O_2_
*M* *_r_* = 268.31
Monoclinic, 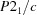

*a* = 7.671 (4) Å
*b* = 13.174 (7) Å
*c* = 13.807 (7) Å
β = 102.680 (7)°
*V* = 1361.3 (12) Å^3^
*Z* = 4
Mo *K*α radiation
μ = 0.09 mm^−1^
*T* = 273 K
0.32 × 0.28 × 0.26 mm

#### Data collection

Bruker SMART APEXII CCD area-detector diffractometer
Absorption correction: multi-scan (*SADABS*; Bruker, 2005 ▶) *T*_min_ = 0.973, *T*_max_ = 0.978
6894 measured reflections
2389 independent reflections
2056 reflections with *I* > 2σ(*I*)
*R*_int_ = 0.086

#### Refinement

*R*[*F* ^2^ > 2σ(*F* ^2^)] = 0.047
*wR*(*F* ^2^) = 0.134
*S* = 1.05
2389 reflections
184 parameters
H-atom parameters constrained
Δρ_max_ = 0.23 e Å^−3^
Δρ_min_ = −0.26 e Å^−3^

### 

Data collection: *APEX2* (Bruker, 2005 ▶); cell refinement: *SAINT* (Bruker, 2005 ▶); data reduction: *SAINT*; program(s) used to solve structure: *SHELXS97* (Sheldrick, 2008 ▶); program(s) used to refine structure: *SHELXL97* (Sheldrick, 2008 ▶); molecular graphics: *SHELXTL* (Sheldrick, 2008 ▶); software used to prepare material for publication: *SHELXTL*.

## Supplementary Material

Crystal structure: contains datablock(s) I, global. DOI: 10.1107/S1600536812040299/rz5006sup1.cif

Structure factors: contains datablock(s) I. DOI: 10.1107/S1600536812040299/rz5006Isup2.hkl

Supplementary material file. DOI: 10.1107/S1600536812040299/rz5006Isup3.cml

Additional supplementary materials:  crystallographic information; 3D view; checkCIF report

## Figures and Tables

**Fig. 1.**
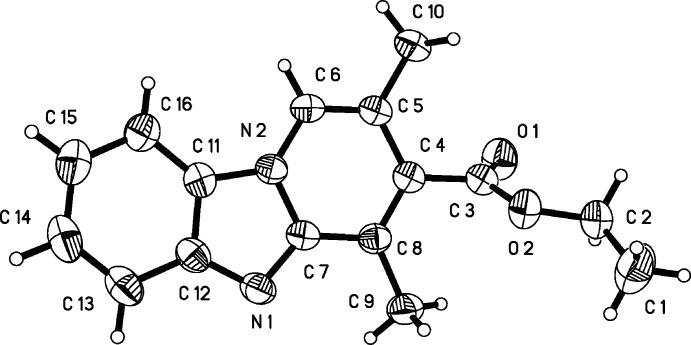
The molecular structure of the title compound, showing displacement ellipsoids drawn at the 50% probability level.

## supplementary crystallographic information

### Comment

The synthesis of nitrogen-containing heterocyclic compounds has been a subject of great interest due to the wide application in pharmaceutical fields (Ge *et al.*, 2009; Ge *et al.*, 2011). Some pyrido[1,2-*a*]benzimidazole derivatives have been of interest for their biological activities, such as antineoplastic activity and central GABA-A receptor modulators for the treatment of anxiety (Badawey *et al.*; 1999).

The fused-ring system of the title compound (Fig. 1) is approximately planar, the dihedral angles formed by the mean planes through the imidazole ring with the dihydropyridine and benzene rings being 1.54 (9) and 1.85 (9)°, respectively. The mean plane defined by the carboxylate group (C3/C4/O1/O2) is tilted by 60.38 (5)° with respect to the plane of the benzene ring. The crystal structure is stabilized only by van der Waals interactions.

### Experimental

To a 50 ml round-bottomed flask were added 1-(1*H*-benzo[*d*]imidazol-2-yl)ethanone (1.00 mmol), (*E*)-ethyl 4-bromobut-2-enoate (2.00 mmol), potassium carbonate (0.28 g, 2.05 mmol) and dry DMF (10 ml). The mixture was stirred at room temperature for 6 h. The solvent was removed under reduced pressure and the product was isolated by column chromatography on silica gel (yield 70%). Crystals of the title compound suitable for X-ray diffraction were obtained by allowing a refluxed solution of the product in ethyl acetate to cool slowly to room temperature (without temperature control) and allowing the solvent to slowly evaporate for 2 d.

### Refinement

All H atoms were placed in geometrically calculated positions and refined using a riding model, with C—H = 0.93-0.97 Å and with *U*_iso_(H) = 1.2*U*_eq_(C) or 1.5*U*_eq_(C) for methyl H atoms. A rotating group model was applied to the methyl groups.

### Crystal data

**Table:**

C_16_H_16_N_2_O_2_	*F*(000) = 568
*M**_r_* = 268.31	*D*_x_ = 1.309 Mg m^−^^3^
Monoclinic, *P*2_1_/*c*	Mo *K*α radiation, λ = 0.71073 Å
Hall symbol: -P 2ybc	Cell parameters from 4261 reflections
*a* = 7.671 (4) Å	θ = 3.0–28.2°
*b* = 13.174 (7) Å	µ = 0.09 mm^−^^1^
*c* = 13.807 (7) Å	*T* = 273 K
β = 102.680 (7)°	Block, colourless
*V* = 1361.3 (12) Å^3^	0.32 × 0.28 × 0.26 mm
*Z* = 4	

### Data collection

**Table:**

Bruker SMART APEXII CCD area-detector diffractometer	2389 independent reflections
Radiation source: fine-focus sealed tube	2056 reflections with *I* > 2σ(*I*)
Graphite monochromator	*R*_int_ = 0.086
phi and ω scans	θ_max_ = 25.0°, θ_min_ = 2.2°
Absorption correction: multi-scan (*SADABS*; Bruker, 2005)	*h* = −5→9
*T*_min_ = 0.973, *T*_max_ = 0.978	*k* = −15→14
6894 measured reflections	*l* = −16→16

### Refinement

**Table:**

Refinement on *F*^2^	Primary atom site location: structure-invariant direct methods
Least-squares matrix: full	Secondary atom site location: difference Fourier map
*R*[*F*^2^ > 2σ(*F*^2^)] = 0.047	Hydrogen site location: inferred from neighbouring sites
*wR*(*F*^2^) = 0.134	H-atom parameters constrained
*S* = 1.05	*w* = 1/[σ^2^(*F*_o_^2^) + (0.075*P*)^2^ + 0.2415*P*] where *P* = (*F*_o_^2^ + 2*F*_c_^2^)/3
2389 reflections	(Δ/σ)_max_ = 0.052
184 parameters	Δρ_max_ = 0.23 e Å^−^^3^
0 restraints	Δρ_min_ = −0.26 e Å^−^^3^

### Special details

**Table:**

Geometry. All e.s.d.'s (except the e.s.d. in the dihedral angle between two l.s. planes) are estimated using the full covariance matrix. The cell e.s.d.'s are taken into account individually in the estimation of e.s.d.'s in distances, angles and torsion angles; correlations between e.s.d.'s in cell parameters are only used when they are defined by crystal symmetry. An approximate (isotropic) treatment of cell e.s.d.'s is used for estimating e.s.d.'s involving l.s. planes.
Refinement. Refinement of *F*^2^ against ALL reflections. The weighted *R*-factor *wR* and goodness of fit *S* are based on *F*^2^, conventional *R*-factors *R* are based on *F*, with *F* set to zero for negative *F*^2^. The threshold expression of *F*^2^ > σ(*F*^2^) is used only for calculating *R*-factors(gt) *etc*. and is not relevant to the choice of reflections for refinement. *R*-factors based on *F*^2^ are statistically about twice as large as those based on *F*, and *R*- factors based on ALL data will be even larger.

### Fractional atomic coordinates and isotropic or equivalent isotropic displacement parameters (Å^2^)

**Table:**

	*x*	*y*	*z*	*U*_iso_*/*U*_eq_	
N1	0.75792 (18)	−0.01609 (10)	0.27023 (9)	0.0440 (4)	
N2	0.72152 (15)	−0.05514 (9)	0.10717 (9)	0.0351 (3)	
O1	1.03253 (17)	0.23943 (10)	−0.01104 (10)	0.0594 (4)	
O2	0.80757 (16)	0.30461 (8)	0.04828 (9)	0.0487 (3)	
C1	0.7586 (4)	0.47805 (16)	0.07813 (19)	0.0802 (7)	
H1A	0.6335	0.4681	0.0505	0.120*	
H1B	0.7907	0.5467	0.0664	0.120*	
H1C	0.7830	0.4654	0.1483	0.120*	
C2	0.8641 (3)	0.40713 (12)	0.03063 (14)	0.0531 (5)	
H2A	0.8429	0.4204	−0.0401	0.064*	
H2B	0.9906	0.4155	0.0590	0.064*	
C3	0.9069 (2)	0.22855 (11)	0.02743 (11)	0.0400 (4)	
C4	0.84263 (19)	0.12847 (11)	0.05743 (10)	0.0365 (4)	
C5	0.78468 (19)	0.05213 (11)	−0.01807 (10)	0.0360 (4)	
C6	0.72718 (19)	−0.03754 (12)	0.00956 (10)	0.0373 (4)	
H6	0.6910	−0.0881	−0.0376	0.045*	
C7	0.77753 (19)	0.01635 (11)	0.18186 (10)	0.0358 (3)	
C8	0.84377 (19)	0.11132 (11)	0.15597 (11)	0.0376 (4)	
C9	0.9153 (2)	0.18433 (13)	0.23890 (12)	0.0505 (4)	
H9A	1.0014	0.2284	0.2196	0.076*	
H9B	0.9714	0.1471	0.2972	0.076*	
H9C	0.8189	0.2241	0.2529	0.076*	
C10	0.7900 (2)	0.07044 (13)	−0.12539 (11)	0.0460 (4)	
H10A	0.7427	0.0123	−0.1643	0.069*	
H10B	0.9113	0.0816	−0.1306	0.069*	
H10C	0.7194	0.1291	−0.1495	0.069*	
C11	0.66408 (19)	−0.13957 (11)	0.15187 (11)	0.0377 (4)	
C12	0.6866 (2)	−0.11231 (12)	0.25233 (11)	0.0410 (4)	
C13	0.6335 (2)	−0.18090 (14)	0.31784 (13)	0.0527 (5)	
H13	0.6463	−0.1652	0.3847	0.063*	
C14	0.5616 (2)	−0.27222 (14)	0.28027 (14)	0.0566 (5)	
H14	0.5252	−0.3183	0.3229	0.068*	
C15	0.5415 (2)	−0.29809 (13)	0.17999 (14)	0.0551 (5)	
H15	0.4928	−0.3607	0.1577	0.066*	
C16	0.5929 (2)	−0.23193 (12)	0.11366 (13)	0.0473 (4)	
H16	0.5805	−0.2484	0.0470	0.057*	

### Atomic displacement parameters (Å^2^)

**Table:**

	*U*^11^	*U*^22^	*U*^33^	*U*^12^	*U*^13^	*U*^23^
N1	0.0471 (8)	0.0517 (8)	0.0327 (7)	−0.0038 (6)	0.0078 (5)	0.0015 (6)
N2	0.0331 (6)	0.0393 (7)	0.0329 (6)	0.0012 (5)	0.0070 (5)	−0.0004 (5)
O1	0.0582 (8)	0.0569 (7)	0.0723 (9)	−0.0017 (6)	0.0340 (7)	0.0058 (6)
O2	0.0493 (7)	0.0397 (6)	0.0601 (7)	0.0010 (5)	0.0186 (5)	0.0031 (5)
C1	0.0948 (17)	0.0504 (11)	0.1022 (18)	0.0057 (11)	0.0363 (14)	−0.0058 (11)
C2	0.0638 (11)	0.0409 (9)	0.0546 (10)	−0.0031 (8)	0.0126 (8)	0.0046 (8)
C3	0.0396 (8)	0.0440 (9)	0.0363 (8)	0.0011 (7)	0.0080 (6)	0.0016 (6)
C4	0.0319 (7)	0.0413 (8)	0.0366 (8)	0.0036 (6)	0.0085 (6)	0.0005 (6)
C5	0.0321 (7)	0.0438 (8)	0.0324 (7)	0.0057 (6)	0.0074 (6)	0.0000 (6)
C6	0.0366 (8)	0.0432 (8)	0.0314 (7)	0.0027 (6)	0.0055 (6)	−0.0043 (6)
C7	0.0333 (7)	0.0421 (8)	0.0318 (7)	0.0019 (6)	0.0064 (6)	−0.0017 (6)
C8	0.0355 (8)	0.0409 (8)	0.0360 (8)	0.0017 (6)	0.0069 (6)	−0.0024 (6)
C9	0.0584 (10)	0.0523 (10)	0.0397 (9)	−0.0096 (8)	0.0084 (7)	−0.0069 (7)
C10	0.0473 (9)	0.0566 (10)	0.0345 (8)	0.0042 (7)	0.0097 (7)	0.0030 (7)
C11	0.0311 (7)	0.0401 (8)	0.0419 (8)	0.0032 (6)	0.0081 (6)	0.0046 (6)
C12	0.0352 (8)	0.0488 (9)	0.0380 (8)	0.0030 (6)	0.0057 (6)	0.0067 (6)
C13	0.0480 (10)	0.0654 (11)	0.0432 (9)	−0.0023 (8)	0.0067 (7)	0.0143 (8)
C14	0.0476 (10)	0.0582 (11)	0.0622 (11)	−0.0028 (8)	0.0085 (8)	0.0237 (9)
C15	0.0480 (10)	0.0450 (9)	0.0711 (12)	−0.0029 (7)	0.0105 (8)	0.0066 (8)
C16	0.0449 (9)	0.0444 (9)	0.0534 (10)	−0.0003 (7)	0.0124 (7)	−0.0015 (7)

### Geometric parameters (Å, º)

**Table:**

N1—C7	1.332 (2)	C6—H6	0.9300
N1—C12	1.381 (2)	C7—C8	1.425 (2)
N2—C6	1.3775 (19)	C8—C9	1.504 (2)
N2—C11	1.3899 (19)	C9—H9A	0.9600
N2—C7	1.393 (2)	C9—H9B	0.9600
O1—C3	1.2065 (19)	C9—H9C	0.9600
O2—C3	1.3283 (19)	C10—H10A	0.9600
O2—C2	1.455 (2)	C10—H10B	0.9600
C1—C2	1.480 (3)	C10—H10C	0.9600
C1—H1A	0.9600	C11—C16	1.389 (2)
C1—H1B	0.9600	C11—C12	1.406 (2)
C1—H1C	0.9600	C12—C13	1.401 (2)
C2—H2A	0.9700	C13—C14	1.377 (3)
C2—H2B	0.9700	C13—H13	0.9300
C3—C4	1.498 (2)	C14—C15	1.401 (3)
C4—C8	1.377 (2)	C14—H14	0.9300
C4—C5	1.446 (2)	C15—C16	1.383 (2)
C5—C6	1.345 (2)	C15—H15	0.9300
C5—C10	1.511 (2)	C16—H16	0.9300
			
C7—N1—C12	104.51 (12)	C4—C8—C9	124.71 (14)
C6—N2—C11	130.57 (13)	C7—C8—C9	117.46 (13)
C6—N2—C7	122.66 (13)	C8—C9—H9A	109.5
C11—N2—C7	106.78 (12)	C8—C9—H9B	109.5
C3—O2—C2	117.24 (13)	H9A—C9—H9B	109.5
C2—C1—H1A	109.5	C8—C9—H9C	109.5
C2—C1—H1B	109.5	H9A—C9—H9C	109.5
H1A—C1—H1B	109.5	H9B—C9—H9C	109.5
C2—C1—H1C	109.5	C5—C10—H10A	109.5
H1A—C1—H1C	109.5	C5—C10—H10B	109.5
H1B—C1—H1C	109.5	H10A—C10—H10B	109.5
O2—C2—C1	107.44 (15)	C5—C10—H10C	109.5
O2—C2—H2A	110.2	H10A—C10—H10C	109.5
C1—C2—H2A	110.2	H10B—C10—H10C	109.5
O2—C2—H2B	110.2	C16—C11—N2	131.97 (15)
C1—C2—H2B	110.2	C16—C11—C12	123.44 (14)
H2A—C2—H2B	108.5	N2—C11—C12	104.56 (13)
O1—C3—O2	123.88 (14)	N1—C12—C13	129.53 (15)
O1—C3—C4	124.72 (14)	N1—C12—C11	111.65 (13)
O2—C3—C4	111.39 (13)	C13—C12—C11	118.81 (15)
C8—C4—C5	122.15 (14)	C14—C13—C12	118.01 (17)
C8—C4—C3	119.11 (13)	C14—C13—H13	121.0
C5—C4—C3	118.72 (13)	C12—C13—H13	121.0
C6—C5—C4	118.31 (14)	C13—C14—C15	122.22 (16)
C6—C5—C10	119.97 (14)	C13—C14—H14	118.9
C4—C5—C10	121.71 (14)	C15—C14—H14	118.9
C5—C6—N2	120.53 (13)	C16—C15—C14	121.04 (17)
C5—C6—H6	119.7	C16—C15—H15	119.5
N2—C6—H6	119.7	C14—C15—H15	119.5
N1—C7—N2	112.50 (13)	C15—C16—C11	116.48 (16)
N1—C7—C8	129.02 (14)	C15—C16—H16	121.8
N2—C7—C8	118.48 (13)	C11—C16—H16	121.8
C4—C8—C7	117.80 (13)		

## References

[bb1] Badawey, E. S. A. M. & Kappe, T. (1999). *Eur. J. Med. Chem.* **34**, 663–667. 10.1016/s0223-5234(00)80036-7 11278052

[bb2] Bruker (2005). *APEX2*, *SAINT* and *SADABS* Bruker AXS Inc., Madison, Wisconsin, USA.

[bb3] Ge, Y. Q., Jia, J., Li, Y., Yin, L. & Wang, J. W. (2009). *Heterocycles*, **78**, 197–206.

[bb4] Ge, Y. Q., Jia, J., Yang, H., Tao, X. T. & Wang, J. W. (2011). *Dyes Pigm.* **88**, 344–349.

[bb5] Sheldrick, G. M. (2008). *Acta Cryst.* A**64**, 112–122. 10.1107/S0108767307043930 18156677

